# Perceived inequity, professional and personal fulfillment by women intensivists in France

**DOI:** 10.1186/s13613-021-00860-2

**Published:** 2021-05-12

**Authors:** Caroline Hauw-Berlemont, Cécile Aubron, Nadia Aissaoui, Laetitia Bodet-Contentin, Florence Boissier, Muriel Sarah Fartoukh, Mercedes Jourdain, Julien Le Marec, Julia Pestel, Charlotte Salmon Gandonnière, Fabienne Tamion, Olfa Hamzaoui, FEMMIR Group for the SRLF Trial Group

**Affiliations:** 1 Médecine Intensive Réanimation, Hôpital Européen Georges Pompidou, AP-HP, Université de Paris, Paris, France; 2 Médecine Intensive Réanimation, Centre Hospitalier Régional Et Universitaire de Brest, Université de La Bretagne Occidentale, Brest, France; 3 Cardiovascular Research Center (PARCC), APHP, Hôpital Européen Georges Pompidou, Université de Paris, INSERM Unit 970, Paris, France; 4 Médecine Intensive Réanimation, INSERM CIC 1415, CRICS-TriGGERSep Network, CHRU de Tours and methodS in Patient-Centered Outcomes and Health ResEarch (SPHERE), Université de Tours, INSERM UMR 1246, Tours, France; 5 Médecine Intensive Réanimation, Hôpital Universitaire de Poitiers, Poitiers, France; 6 INSERM CIC 1402 (ALIVE Group), Université de Poitiers, Poitiers, France; 7 Service de Médecine Intensive Réanimation, Faculté de Médecine Sorbonne, Hôpital Tenon, APHP, and APHP, Sorbonne Université, Paris, France; 8 Médecine Intensive Et Réanimation - CHU de Lille, Lille, France; 9 Membre de L’unité INSERM U1190 - Recherche Translationnelle Sur Le Diabète, Lille, France; 10 AP-HP Sorbonne Université, Site Pitié-Salpêtrière Charles Foix, Service de Pneumologie, Médecine Intensive - Réanimation, Département R3S; Sorbonne Université, INSERM, UMRS1158 Neurophysiologie Respiratoire Expérimentale et Clinique, Paris, France; 11 Diplôme D’étude Spécialisée Médecine Intensive Réanimation (DESMIR), Inter-région Sud-Ouest, France; 12 Médecine Intensive Réanimation, INSERM CIC 1415, CRICS-TriGGERSep Network, CHRU de Tours, Tours, France; 13 Médecine Intensive Et Réanimation, Hôpital Universitaire de Rouen, Rouen, France; 14 INSERM U1096 EnVi, Université Normandie, UNIROUEN, caen, France; 15 Université Paris-Saclay, AP-HP, Hôpital Antoine Béclère, Service de réanimation polyvalente, Clamart, France

**Keywords:** Quality of life, Work–life balance, Women in medicine, Intensive care, Women intensivists

## Abstract

**Background:**

The medical workforce has been feminized for the last two decades worldwide. Nonetheless, women remain under-represented among intensivists. We conducted a survey among French women intensivists to assess their professional and personal quality of life and their perception of potential gender discrimination at work.

**Methods:**

We conducted an observational descriptive study by sending a survey, designed by the group FEMMIR (FEmmes Médecins en Médecine Intensive Réanimation), to women intensivists in France, using primarily the Société de Réanimation de Langue Française (SRLF) mailing list. The questionnaire was also available online between September 2019 and January 2020 and women intensivists were encouraged to answer through email reminders. It pertained to five main domains, including demographic characteristics, work position, workload and clinical/research activities, self-fulfillment scale, perceived discrimination at work and suggested measures to implement.

**Results:**

Three hundred and seventy-one women responded to the questionnaire, among whom 16% had an academic position. Being a woman intensivist and pregnancy were both considered to increase difficulties in careers’ advancement by 31% and 73% of the respondents, respectively. Almost half of the respondents (46%) quoted their quality of life equal to or lower than 6 on a scale varying from 1 (very bad quality of life) to 10 (excellent quality of life). They were 52% to feel an imbalance between their personal and professional life at the cost of their personal life. Gender discrimination has been experienced by 55% of the respondents while 37% confided having already been subject of bullying or harassment. Opportunities to adjust their work timetable including part-time work, better considerations for pregnant women including increasing the number of intensivists and the systematic replacement during maternity leave, and the respect of the law regarding the paternity leave were suggested as key measures to enable better professional and personal accomplishment by women intensivists.

**Conclusion:**

In this first large French survey in women intensivists, we pointed out issues felt by women intensivists that included an imbalance between professional and personal life, a perceived loss of opportunity due to the fact of being a woman, frequent reported bullying or harassment and a lack of consideration of the needs related to pregnancy and motherhood.

## Introduction

Medicine has been living a profound transformation for the last two decades, with the feminization of the medical workforce worldwide. In France in 2018, 59% of the new medical board registrations were signed by women [1] and 46.8% of medical specialists were women in 2015 vs 40.8% in 2010 [2]. According to a recent study by Venkatesh et al., inadequate gender disparity in intensive care units (ICUs) remains, women comprising between 20 and 50% of the critical care medicine workforce, depending on the geographic region [3]. In France, 23% of medical intensivists are women [2]. In a French survey conducted among hospital practitioners regardless of their specialty, 43% of women doctors felt gender discrimination vs 18% of men; 36% of women declared to have modified their professional career because of household burden; and 69% of women thought they would have had a different career being a man, highlighting the perception of the impact of gender on professional fulfillment [4]. There is no data available for women intensivists specifically. Intensive care is a demanding specialty with common long day of work and many night shifts. In addition, younger doctors might perceive intensive care as a stressful and constraining specialty incompatible with personal life fulfillment. To know better what the real perception of professional and personal fulfilment in women intensivists was, we conducted a study to assess women intensivists’ quality of life at work and of personal life, as well as perception of gender equality at work and to identify measures that could help improving professional and personal fulfillment of this population.

## Material and methods

This descriptive observational quantitative study was designed, coordinated and executed by the FEmmes Médecins en Médecine Intensive Réanimation (FEMMIR), a group of women intensivists members of the French Intensive Care Society-Société de Réanimation de Langue Française (SRLF). The Equator Network guidelines were used for conducting and reporting the results of this observational study [5].

### Questionnaire development

Between June and August 2019, the questionnaire was designed by three members of the group. We conducted a draft revision reviewed by all the members of the group FEMMIR. Some questions were reworded or added. Finally, the questionnaire (annexe 1) comprised 41 questions, distributed into five main domains: demographics and private life characteristics, work position and activities, how being a woman intensivist impacts on career advancement and quality of life, perceived discrimination at work, suggestions to improve female intensivists work conditions. Twenty questions were yes–no ones, twenty items were evaluated on a 5-point Likert scale and one was an open-response question. The questionnaire was organized around three themes: women working conditions, gender inequity perception and perceived quality of life.

### Distribution

In September 2019, after the approval of the SRLF executive board, the electronic link to the self-administered questionnaire was sent to 732 (33%) women doctors among 2104 French-speaking, university and non-university affiliated doctor intensivists through the SRLF mailing list. It was specified in the email that it was intended to women intensivists and recipients were encouraged to invite female intensivist colleagues, who might not have been a SRLF member, to answer the questionnaire. The survey was also spread on social networks and last, all the SRLF members received the electronic link through the bi-monthly SRLF newsletter. A monthly reminder was sent between September 2019 and January 2020, to increase the participation rate. Moreover, a link to the questionnaire was available on the SRLF website and it was accessible online from September 15, 2019 to January 30, 2020. The SurveyMonkey© platform was used. The survey required approximately 5 to 10 min to be completed. The participation was anonymous and voluntary. We offered no compensation for survey participation.

### Statistical analysis

We present descriptive statistics characterizing demographics and closed-ended questions. The open-response question was analyzed by three members of the group according to a semi-quantitative method, leading to classify answers into five categories based on their theme and the frequency they were proposed by the respondents.

All data were analyzed using SurveyMonkey©’s online basic statistics.

## Results

### Respondents’ private life characteristics

Altogether, 371 women intensivists responded to the survey. More than half of them (*n* = 220, 59.3%) were between 30 and 40 years old and only 8% were older than 50 years. Fifty-six percent (207/371) had no children and among the 162 women intensivists who had children, 78% of them had a maximum number of two children (Table 1).

**Table 1:** General characteristics of the respondents

	n	Percentage
Age distribution (*n* = 371)		
< 30 years	72	19.41%
Between 30 and 40	218	58.76%
Between 40 and 50	51	13.75%
Between 50 and 60	25	6.74%
> 60 years	5	1.35
Family situation (*n* = 371)		
Civil partnership/married	167	45.01%
Couple	100	26.95%
Single	90	24.26%
Divorced	13	3.50%
Other situation	1	0.27%
Children (*n* = 371)		
Yes	164	44.20%
No	207	55.80%
How many children(*n* = 162)		
1	54	33.33%
2	73	45.06%
3 or more	35	21.60%

### Work positions and activities

Most of the respondents (84%) had a non-academic position. The daily mean number of hours spent at work was 10.2 h (SD 2.2) (Table 2).

**Table 2:** Workload main characteristics (298 respondents)

Variables	
Working hours per a day, mean (SD)	10 (2.2)
Working hours per week	
Between 20 and 40 h	38 (12.7%)
Between 40 and 60 h	183 (61.4%)
> 60 h	74 (24.8%)
End of the day time, mean (SD)	7 pm ( 0.58)
Night shift a month	
Between 3 and 4	120/298 (40.3%)
> 4	158/298 (53%)
Work on the weekend in addition to night shift	
0	118 (39.6%)
1 to 2	108 (36.2%)
3 or 4	36 (12.1%)
Time dedicated to research	
0 h /week	198/298 (66%)
10 h /week	85/298 (28.5%)

Ninety-three percent of the respondents (278/298) worked more than 3 night shifts per month and they were 60% (180/298) to work on the weekend in addition to their nightshift and daily activity. Altogether, they worked either between 40 and 60 h (51% of the 298 respondents to this question) or more than 60 h (48% of the 298 respondents to this question) a week.

Time at work was mainly dedicated to patient care, i.e., clinical activities (86% of the respondents) (Table 3). The time dedicated to research was different according to the academic status of the respondents. Seventy percent of the respondents with a non-university status had no time for research, because their time was mainly dedicated to clinical activities. Of note, almost 40% of the university respondents declared having no time for research.

**Table 3:** Time allocated to research according to university status of the respondents

	Near Zero *n* (%)	Around 10 h *n *(%)	Around 20 h *n* (%)
Non-university status	173 (71)	65 (27)	5(2)
University status	18 (39)	20 (43)	6 (13)
Private clinics	23 (85)	4 (15)	0 (0)

Seventy-seven percent of the respondents declared working at home after a working day (49% occasionally and 28% routinely) regardless of their academic status.

### Career advancement and quality of life

Although 43% of the respondents felt supported to conduct research, 31% considered that being a woman intensivist increased difficulties in academic and careers’ advancements. One third of the respondents (90/298) felt they had been disadvantaged because of their sex and declared that their work conditions would have been better being a man (Table 4).

**Table 4:** Impact of gender in career advancement and quality of life

Question	*n*/total of respondents to this question (%)
Being a woman is a barrier in the intensivist career’s advancement	90/298 (30%)
Pregnancy is a barrier to academic advancement of women intensivists	201/276 (79%)
There is an imbalance between personal and professional life	75/276 (27%)
Wish they could work part-time	136/276 (49%)

**Fig. 1:**
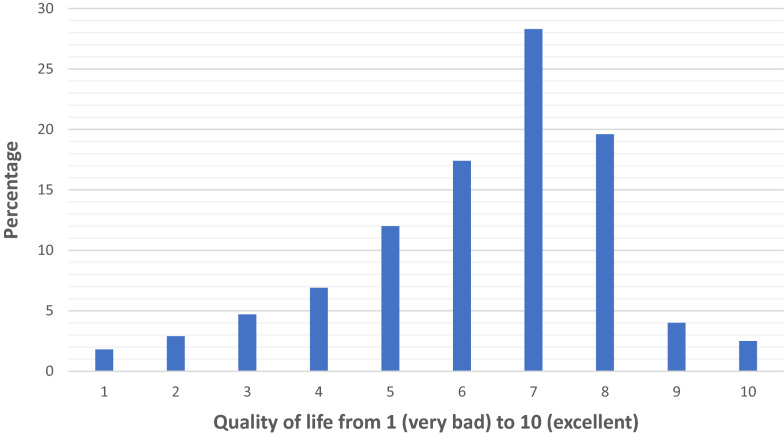
Represents the distribution of the respondents as a function of their perceived quality of life at work, on a scale varying from 1 (very bad quality of life) to 10 (excellent quality of life)) (*N* = 276)

**Fig. 2:**
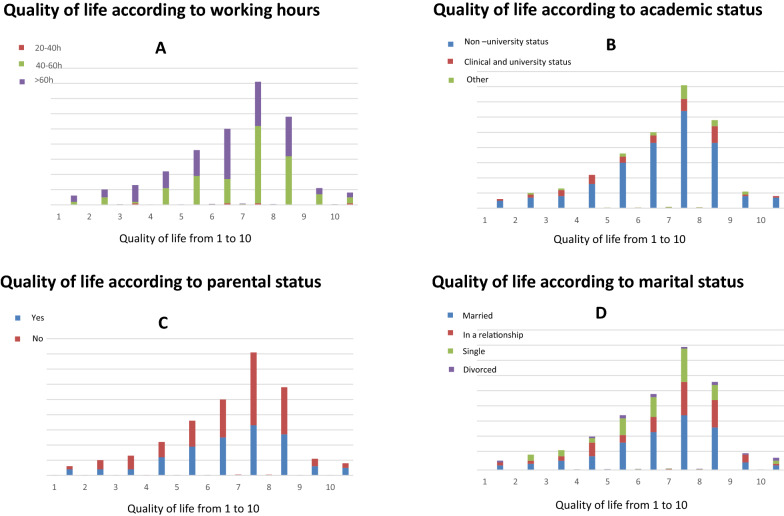
The evaluation of quality of life according to the numbers of working hours (**a**), their academic status (**b**), marital status (**c**) and parental status (**d**)

**Fig. 3:**
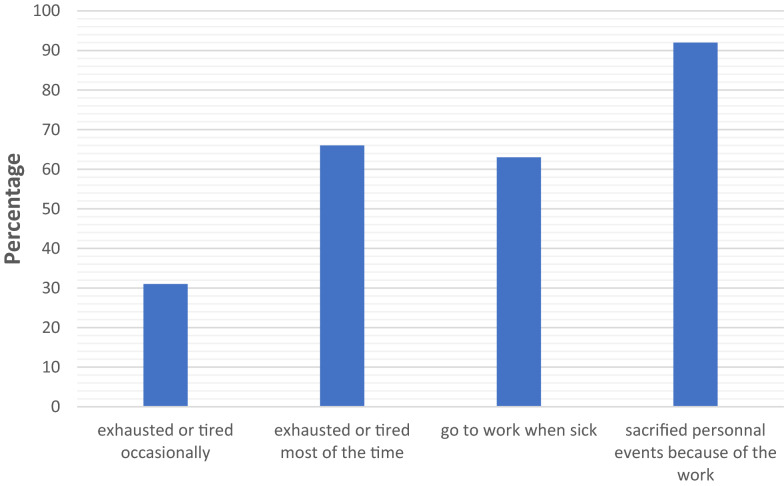
Represents the distribution of the respondents as a function of their perceived fatigue and in function of being sick or not (*N* = 276)

Almost half of the respondents (46%) quoted their quality of life equal to or lower than six on a scale varying from 1 (very bad quality of life) to 10 (excellent quality of life) (Fig. 1). The evaluation of quality of life was different according to the numbers of working hours, and their academic, parental and marital status (Fig. 2). Sixty-one percent (169/276) declared that their quality of life at work could be improved if their own needs were considered. Indeed, 51.7% (75/276) reported that they had an imbalance between personal and professional life and 21% of the respondents confessed not having any children because their job was not compatible with motherhood. Forty-nine percent (136/276) would have preferred working part-time, and more than two-thirds (201/276) considered pregnancy as a barrier to a woman intensivist career advancement. All the respondents (268/276, 97%) felt tired or exhausted (Fig. 3).

### Perceived discrimination at work

Discrimination has been experienced by 55% of women intensivists (125/276), including discrimination at the bedside with male colleagues, patients or patients’ families or with the nurse team for 25%, 20%, and 10% of them, respectively. Respondents gave examples of discriminations they have been experiencing 1) women felt their men colleagues doubted of women intensivists’ performances and that they were less consulted for important decisions regarding team management 2) with patients and families, who often asked to talk to a man to have medical information or turn to the man in the room when they wanted to talk to a doctor 3) with nurses, women doctors felt they needed to prove themselves more than men colleagues.

Thirty-seven percent (101/276) of the respondents confided having already been subject of bullying or harassment. Among them, 56% were victims of sexual harassment, 77% reported moral harassment and 36% both (Table 5).

**Table 5:** Humiliation and bullying in the workplace

Variables	
Humiliation in the workplace, n (%)	125/276 (45.3)
Bullying in the workplace, n (%)	101/276 (36.6%)
Sexual harassment	55/99 (56%)
Moral harassment	44/99 (44%)

### Suggestions to improve women intensivists work conditions

One hundred and eleven respondents made one or more propositions to improve their work conditions. These suggestions have been classified into five groups based on their theme and the frequency they were proposed (Table 6). The different suggestions were: 1) adjusting working hours (including no planned meeting after 6 pm), but not necessarily working less and the possibility to work part-time when needed to allow a better balance between their personal and professional life; 2) increasing the number of intensivists per ICU, which would allow replacement of intensivists during maternity leave or prolonged sick leave and would also contribute to a better quality of life at work (with a constant work load and night shifts number along the year including holidays); 3) exempting pregnant intensivists from night shift; 4) supporting a mandatory paternity leave; 5) providing support for breast feeding to mothers who wish it; 6) enhancing child care for children of intensivists; 7) banning sexism at work to build a safer work environment for all intensivists.

**Table 6:** Top 5 of the suggestions made by 111 women intensivists to improve their work conditions

Suggestions	Respondents^a^ n (%)
Allow part-time—adapt working hours	44 (39.6)
Increase number of intensivists per ICU	15 (13.5)
Measures related to maternity and paternity^b^	21 (19)
Improve childcare availability for children of intensivist	18 (16.2)
Ban gender harassment	15 (13.5)

^a^One respondent can make several propositions

^b^Replacement of female intensivist while in maternity leave, no night shift when pregnant, allow male intensivist to get their paternity leave

## Discussion

### Study key findings

This is the first large French questionnaire dealing with women physicians’ work conditions in medical intensive care specialty. Being a woman was considered to increase difficulties in careers or academic advancement of women intensivists by one-third of the respondents and two-thirds think that pregnancy was a barrier to their career‘s advancement. Almost half of the respondents quoted their quality of life equal to or lower than six on a scale varying from 1 (very bad quality of life) to 10 (excellent quality of life) while they were 52% to feel an imbalance between their personal and professional life at the cost of their personal life. Gender discrimination has been experienced by 55% of the respondents. Finally, among the five main measures proposed to improve quality of life at work of women intensivists, part-time work was the most frequently suggested.

### Comparison with the literature

Studies from other areas of medicine have reported gender-based differences in career advancement [6], leadership positions [7] and byline positions in case of dual first authorship [8]. In our specialty, as observed in other medical fields, women are less represented among academic positions. Although 16% of the respondents declared having an academic position (tenured and non-tenured), only 7.8% of the tenured full professors in Medical Intensive Care in France were women at the time of the survey according to the Collège des Enseignants de Médecine Intensive Réanimation (CeMIR) (French College of Medical Intensive Care). These findings are in accordance with the rate of 9% of women Anesthesia and Intensive Care full professors previously reported by Godier et al*.* [9]. The Association of American Medical College showed that the gap between women and men advancement still existed in 2019 [10] and a large recent study reported this gap has not been narrowing over time [11]. In our study, one-third of the women intensivists (34%) felt they had been disadvantaged because of their gender. A previous survey of the College of Intensive Care Medicine of Australia and New Zealand found that among the 127 female fellow respondents, 37% felt similar inequity [12]. Those findings together with other published data support the theory of biases that impede women’s advancement in what are perceived as historically male-dominant fields [13, 14]. Unconscious biases have been argued as one of the main reasons for the gender inequity in numerous domains in medicine and sciences, such as awarded research grant and research funding [15, 16].

Another striking finding of our study is that more than the third of our respondents reported being faced with bullying and sexual harassment. Similar results were reported in a survey of trainees and fellows of the College of Intensive Care Medicine of Australia and New Zealand with an overall prevalence of bullying, discrimination and sexual harassment of 32%, 12% and 3%, respectively. Women reported a significantly greater prevalence of sexual harassment and discrimination than men did [17]. The prevalence of bullying, in a survey from New Zealand’s public health system including senior doctors and dentists [18] was of 38% (at least one negative act on a weekly or daily basis). Prevalence of bullying was associated with high workloads and low peer and managerial support. There were significant differences in rates of bullying by specialty with emergency medicine reporting the highest bullying prevalence (47.9%). The consequences of workplace bullying have been described as the most ‘destructive phenomenon plaguing medical culture’ [19], proved as significant risks to patient safety and quality of patient care [20], staff morale and job satisfaction [21], and the physical and psychological wellbeing of doctors and their coworkers [22, 23]. In France, intensivists are exposed to a high workload and night shifts (half of the respondents work more than 60 h) contributing to increase bullying susceptibility [18].

This workload, the risk of harassment and the feeling that working in intensive care is not compatible with motherhood reflect the hard work conditions in intensive care medicine and may partly explain the hesitation for young doctors to commit in this specialty in general. Our results are different from those reported in previous surveys in other countries perhaps because of the frequent use of part-time contracts, in particular for mothers [18]. Indeed, in France, part-time work in intensivists remains unusual. According to the Centre National de Gestion, in January 2020, only 1.4% of doctors in ICU worked part-time, while in some other medical specialties, up to 50% of the doctors did so [24].

Our questionnaire, beyond being a real-life picture of women’s work conditions in intensive care medicine, should encourage all the intensive care workforce community to display urgent solutions to enhance gender equality in our specialty. Our group FEMMIR, created 18 months ago within the SRLF, has as main objective and mission of improving women work conditions and lives (https://www.srlf.org/projets/femmir/) and increasing women representativeness within the specialty. Indeed, according to the Centre National de Gestion, in 2017, 42.2% of the new residents in medical intensive care medicine were women while 57.5% of all residents were [25]. Our organization among others is calling for variable actions such as the introduction of flexible training policies (including part-time and interrupted training, and parental leave options), guidelines for professional standards of behavior and for appropriate gender equality at various events, and promotion of diversity and equality (guidelines committee, editorial board, education, academics). In addition, FEMMIR initiated habit-changing educational interventions, which are known helping to breach gender bias and change climate [26]. Last, equity and gender biases are not perceived in the same way by doctors depending on their gender and their age [27], which calls for actions that can reach everybody to make them fully aware of what women perceive at work.

### Strengths and limitations

This is the first French survey on perception of women intensivists about discrimination among intensivists. Our study is a current snapshot of the female physician workforce in France. We used the well-established SRLF members’ mailing list including the largest population of intensive care medicine workforce in France and then cover a big number of intensive care units. Our questionnaire included one open-question leaving the opportunity to respondents to express their own opinions and opening to concrete measures to change the ICU environment for women intensivists.

Our questionnaire has some limitations. We assume that our list of women intensivists is not exhaustive. In France, doctors might have different training curricula before becoming intensivists (medical specialty or anesthesia); a proportion of intensivists are not board registered as such but as medical specialists or anesthesiologists. Based on the membership of the SRLF, there are 732 women for 2104 intensivists (34.8%). However, the exact rate of women intensivists in France is unknown as some of them might not be members of the SRLF.

Therefore, 1) some women may not have received the link to the survey if they were not SRLF members; 2) the questionnaire was also accessible to a proportion of women not registered in our national society database via a link on our website, which precludes to provide an exact response rate; 3) and finally, women intensivists working in surgical ICUs are commonly not members of the SRLF.

As the aim of our survey was to draw a snapshot of women intensivists’ work conditions, we did not include men in our panel of respondents purposely; this precluded any comparison between men and women responses, which is a limitation in particular when addressing the issue of quality of life. Furthermore, we cannot discuss the perception of men intensivists regarding their women colleagues’ perceptions on inequity. Our results are per definition based on the respondent’s declaration and might differ from reality; however, this is the limitation of any survey. Respondents could skip some questions leading to some biases and over or underestimations. Indeed, the numbers of respondents vary over the survey 1) some women intensivists may not have been concerned by some questions depending on their status at the time of the survey (i.e., maternity leave, research mobility, or PhD preparation); 2) some of the respondents might not have yet faced some situations and/or difficulties and thus, were not concerned by questions such as the experience of pregnancy or career advancement. Last, in order to assure the completeness of the survey, we had to make choices about data we wanted to collect. Some interesting information is missing, including the type of hospital (university and non-university hospitals).

## Conclusions

In this first large French survey on work life perception and discrimination in women intensivists, we pointed out important problems that women doctors encounter when working in intensive care units, including an imbalance between professional and personal life, a perceived loss of opportunity due to the fact of being a woman, frequent moral and/or sexual harassment and a lack of consideration of the needs related to pregnancy and motherhood. There is an urgent need to engage with the national colleges, trainees and certifying bodies to promote women enrollment into training programs, to facilitate part-time position when needed and to work with employers to develop policies that promote women intensivists careers and a safer workplace.
